# Mice deficient in the mitochondrial branched-chain aminotransferase (BCATm) respond with delayed tumour growth to a challenge with EL-4 lymphoma

**DOI:** 10.1038/s41416-018-0283-7

**Published:** 2018-10-15

**Authors:** Elitsa A. Ananieva, Joshua N. Bostic, Ashley A. Torres, Hannah R. Glanz, Sean M. McNitt, Michelle K. Brenner, Michael P. Boyer, Adele K. Addington, Susan M. Hutson

**Affiliations:** 1 Department of Biochemistry and Nutrition, Des Moines University, 3200 Grand Avenue, Des Moines, IA 50312 USA; 2 Department of Human Nutrition, Foods, and Exercise, Virginia Tech, Integrated Life Sciences Building 0913, 1981 Kraft Drive Blacksburg, Blacksburg, VA 24060 USA; 3 Present Address: Centre for Earth Evolution and Dynamics, University of Oslo, N-0315 Oslo, Norway

**Keywords:** Cancer, Cancer metabolism

## Abstract

**Background:**

The mitochondrial branched-chain aminotransferase (BCATm) is a recently discovered cancer marker with a poorly defined role in tumour progression.

**Methods:**

To understand how a loss of function of BCATm affects cancer, the global knockout mouse BCATmKO was challenged with EL-4 lymphoma under different diet compositions with varying amounts of branched-chain amino acids (BCAAs). Next, the growth and metabolism of EL-4 cells were studied in the presence of different leucine concentrations in the growth medium.

**Results:**

BCATmKO mice experienced delayed tumour growth when fed standard rodent chow or a normal BCAA diet. Tumour suppression correlated with 37.6- and 18.9-fold increases in plasma and tumour BCAAs, 37.5% and 30.4% decreases in tumour glutamine and alanine, and a 3.5-fold increase in the phosphorylation of tumour AMPK in BCATmKO mice on standard rodent chow. Similar results were obtained with a normal but not with a choice BCAA diet.

**Conclusions:**

Global deletion of BCATm caused a dramatic build-up of BCAAs, which could not be utilised for energy or amino acid synthesis, ultimately delaying the growth of lymphoma tumours. Furthermore, physiological, but not high, leucine concentrations promoted the growth of EL-4 cells. BCATm and BCAA metabolism were identified as attractive targets for anti-lymphoma therapy.

## BACKGROUND

The BCAAs, leucine, isoleucine, and valine, have received substantial scientific interest in recent years with respect to their role in tumour progression.^1^ Historically known to influence muscle protein synthesis, insulin resistance, and brain amino acid uptake, BCAAs are also considered important sources of carbon metabolites, nitrogen, and energy for cancer growth.^1–5^ The enzymes catalysing the first step in BCAA degradation (cytosolic and mitochondrial branched-chain aminotransferases, BCATc and BCATm, respectively) are now recognised as prognostic tumour markers in several cancer types.^6–11^ These enzymes catalyse the reversible transamination of BCAAs, which comprises a transfer of the α-amino group of a BCAA to α-ketoglutarate to form glutamate and the respective branched-chain α-keto-acid (BCKA).^12,13^ Extraction of nitrogen from BCAAs for the synthesis of glutamine and nucleotides was reported in lung and pancreatic tumours, as well as, glioblastomas.^7,14,15^ The release of BCKAs, on the other hand, had antiproliferative effects on blast crisis chronic myeloid leukaemia.^16^

BCATc, also known as BCAT1, represents the major BCAT isoenzyme implicated in tumour progression. In fact, BCATc is investigated in many cancer types, although, the effects of BCATc are influenced by the tissue of origin and the oncogenic mutations.^7,9,16–20^ BCATm, however, is not nearly as well explored in cancer. One obstacle in deciphering the role of BCATm in cancer progression and its therapeutic applications is that BCATm is constitutively expressed in all organs, except hepatocytes, while BCATc is expressed primarily in the central nervous system, immune, and cancer cells.^1,21,22^ Nevertheless, recent reports have suggested that BCATm is required for tumour growth in non-small cell lung carcinoma (NSCLC), pancreatic ductal adenocarcinoma (PDAC), and gliomas.^15,23,24^

The emerging interest in oncogenic BCAA metabolism via BCATm prompted us to investigate the role of BCATm in tumour progression. For this purpose, we utilised the global BCATmKO mouse model that was challenged with T cell lymphoma.^25^ While BCAAs, especially leucine, are critical for the activation of T lymphocytes and BCAT isoenzymes are part of a negative feedback regulation of T lymphocyte function, the role of BCAT metabolism in cancerous T lymphocytes has not been explored.^21,26^ In this study, we challenged the BCATmKO mice with the murine EL-4 lymphoma and monitored tumour growth under different BCAA diet formulations. Dramatic build-up of BCAAs had beneficial effects on tumour prevention when BCATmKO mice were fed standard rodent chow or a normal BCAA diet. However, moderately elevated BCAAs in BCATmKO mice, fed a choice BCAA diet, did not reduce tumour growth. In vitro studies further demonstrated that physiological, but not high, leucine concentrations promoted the growth and metabolism of EL-4 cells.

## MATERIALS AND METHODS

### Antibodies and reagents

Antibodies against the eukaryotic translation initiation factor 4E binding protein 1 (4EBP-1), P-4EBP-1 (Thr37/46), the ribosomal protein S6 (S6), P-S6 (Ser240/244), β-tubulin, AMPKα, P-AMPKα (Thr172), lactate dehydrogenase A (LDH-A), P-LDH-A (Tyr10), hexokinase II (HEXII), B-cell lymphoma 2 (Bcl-2), and Bcl-2-associated X (BAX) were purchased from Cell Signalling Technologies (Danvers, MA). Antibodies against BCATm and BCATc were previously described.^5,27^ Rapamycin and *N*-acetyl-leucine amide (NALA) were purchased from LC Laboratories (Woburn, MA) and BACHEM (Bubendorf, Switzerland), respectively. BAX-inhibiting peptide V5 (Bax-V5), BAX-inhibiting negative control peptide (NCP), and Hoechst 33258 were purchased from MilliporeSigma (Burlington, MA). Etoposide was purchased from Enzo Life Sciences (Farmingdale, NY).

### Mice and diets

All animal experiments were approved by the Institutional Animal Care and Use Committee at Virginia Tech. Wild type (WT) and BCATmKO mice (C57BL/6 background) were housed in a temperature-controlled room with a 12-h light/12-h dark cycle and provided free access to water and a choice between normal and low BCAA diets until 12–15 weeks of age. The combination of these two diets is referred to as the choice BCAA diet. The normal BCAA diet contained 12, 8, and 8 g/kg leucine, isoleucine, and valine, while the low BCAA diet contained 0.3, 0.2, and 0.2 g/kg of these amino acids, respectively (Supplementary Table 1). Male and female mice were used in a pilot cancer study with essentially the same results (data not shown), after which, a single gender (females) was used.

### Cell cultures and treatments

Murine EL-4 lymphoma cells, isolated from a mouse T-lymphoma, were purchased from ATCC (Manassas, VA) and maintained in a standard RPMI-1640 medium (CellGro, Manassas, VA), supplemented with 10% FBS (HyClone, UT), and 100 IU/ml of penicillin and 100 µg/ml of streptomycin (HyClone, UT) in a tissue culture incubator at 37 °C and 5% CO_2_. For leucine concentration-gradient experiments, 16 × 10^6^ EL-4 cells were incubated in a custom made BCAA-free RPMI-1640 medium (11-PB-078, CellGro, Manassas, VA), supplemented with 380 µM isoleucine, 171 µM valine, 10% FBS and penicillin/streptomycin solution. The cells were then separated into four cultures (4 × 10^6^ cells/culture) and subsequently treated with 0, 190, 380, and 1140 µM of leucine (0x, 0.5x, 1x, and 3x leucine concentrations used in a standard RPMI-1640 medium) for 48 h. To avoid a single essential amino acid deprivation in EL-4 cells treated with 0 µM leucine, FBS was not dialysed prior to addition to the BCAA-free growth medium. Thus, 10% FBS contributed additional 18 µM leucine, 15.3 µM isoleucine, and 22.5 µM valine as determined by HPLC analysis.^28^ For NALA and rapamycin treatments, a separate set of 16 × 10^6^ EL-4 cells were incubated in a standard RPMI-1640 medium (containing 380 µM leucine) with 10% FBS, as above, and penicillin/streptomycin solution, and after separation into 4 cultures (4 × 10^6^ cells/culture), the cells were left untreated or treated with NALA (10 and 20 mM) or rapamycin (100 nM) for 24 h, respectively. At the end of each treatment, cell growth was determined by the trypan blue dye exclusion method.^29^ Next, EL-4 cells were washed twice with DMEM medium (CellGro, Manassas, VA), pelleted at 1200 rpm, for 7 min, at room temperature, re-suspended in DMEM medium, and used to measure glycolytic and oxidative metabolism (see below). Alternatively, EL-4 cells were washed twice with ice-cold PBS buffer, pelleted at 13,000 rpm, for 5–10 min, at 4 °C, and stored at −80 °C until western blotting as described below.

### In vivo tumour studies under different BCAA diets

#### Tumour induction in mice fed standard rodent chow

WT and BCATmKO mice, between 12 and 15 weeks of age, were switched from a choice BCAA diet to standard rodent chow (day 1) (Harlan-Teklad 2018, Supplementary Table 2). All mice were fasted for 12 h on day 2 and blood was collected for amino acid analysis. On day 3, mice received s.c. injections of 5 × 10^5^ EL-4 cells in 100 µl sterile PBS buffer, on the upper back (WT and BCATmKO tumour-injected, *n* = 9 mice/group). As controls, age-matched WT and BCATmKO mice received 100 µl of a sterile PBS buffer (WT and BCATmKO vehicles, *n* = 6 mice/group). Next, mice were caged individually, and food intake and body weight were measured every other day. Mice were monitored daily for the appearance of tumours and as soon as tumours were visible, tumour volume was measured every day with a digital caliper (formula for tumour volume: *V* = length × (weight)^2^/2). All mice were sacrificed on day 13 by cervical dislocation, following a 12 h fast, unless the tumour size reached a volume of 1 cm^3^ before day 13, in which case animals were terminated earlier. Tumour, heart, kidneys, muscle, and spleen were weighed and stored at −80 °C. Plasma was collected after centrifugation at 4500 rpm for 10 min at 4 °C and stored at −80 °C.

#### Tumour induction in mice fed choice and normal BCAA diets

This study was performed in a similar manner, as above, with the following differences: mice were separated into two groups. One group of mice remained on a choice BCAA diet, while the other group was offered a normal BCAA diet only (Supplementary Table 1). Three days later, mice were s.c. injected with 100 µl sterile PBS buffer with or without 2.5 × 10^5^ EL-4 cells (*n* = 3–9 mice) forming eight groups of experimental mice (WT and BCATmKO tumour-injected and vehicles on choice or normal BCAA diets, respectively). Because the number of cancer cells was reduced by half, tumours appeared around day 9 and the tumour study continued until day 14.

### Amino acid analysis

Amino acid concentrations in tumour tissues and plasmas from WT and BCATmKO mice were measured by HPLC after *O*-phthaldialdehyde derivatisation on a Supelcosil^™^ LC-18 column (15 cm × 4.6 mm, 3 μm) (Sigma, St. Louis, MO) as described.^30^

### Western blotting

Protein was extracted from ground tumour tissues or EL-4 cell pellets, followed by determination of a total protein and Western Blotting as described.^21^ Image J software was used to quantify protein bands (BCATc, BCATm, HEXII, Bcl-2, BAX) and normalise to a loading control (β-tubulin) or to calculate the ratio between phosphorylated and total concentrations of 4EBP-1, S6, AMPKα, and LDH-A.^31^

### Microscopic imaging and chromatin condensation

EL-4 cells treated with either 190 or 1140 µM leucine were simultaneously treated with 100 µM BAX-V5 or 100 µM negative control peptide (NCP) for 48 h. Alternatively, EL-4 cells were grown in the presence of 30 µM etoposide for 24 h. At the end of each treatment, live cells were incubated with 5 µg/ml Hoechst 33258 for 30 min at 37 °C, and fluorescent microscopy to monitor chromatin condensation as a readout of apoptosis was used as described.^32,33^ The percentage of nuclei containing condensed chromatin was determined by dividing the number of brightly stained, small nuclei by the total number of nuclei. At least 100 nuclei were counted for each data point.

### Glycolytic and oxidative metabolism of EL-4 cells

Glycolytic and oxidative metabolism were measured by a Seahorse XF24 Extracellular Flux Analyser (Agilent, Santa Clara, CA) in EL-4 cells grown in the absence or presence of different concentrations of leucine for 48 h or in EL-4 cells treated with 0 and 10 mM NALA for 24 h. Measurements of the basal extracellular acidification rate (ECAR), basal glycolytic rate, and maximum glycolytic capacity were performed as described.^21^ For oxidative metabolism, the oxygen consumption rate (OCR) was measured before and after the addition of oligomycin (a complex V inhibitor), FCCP (a protonophore uncoupler), and antimycin A and rotenone (inhibitors of complex III and I, respectively) as described.^34^

### Statistical analysis

One-way ANOVA was used to assess the difference between WT and BCATmKO (vehicle- and tumour-injected) mice, as well as between EL-4 cells treated with different concentrations of leucine, NALA, rapamycin, BAX-V5, or etoposide. Values are means ± SEM and *P* ≤ 0.05 was considered statistically significant.

## RESULTS

### BCATmKO mice display delayed tumour growth and smaller tumour sizes when challenged with EL-4 lymphoma

**Fig. 1:**
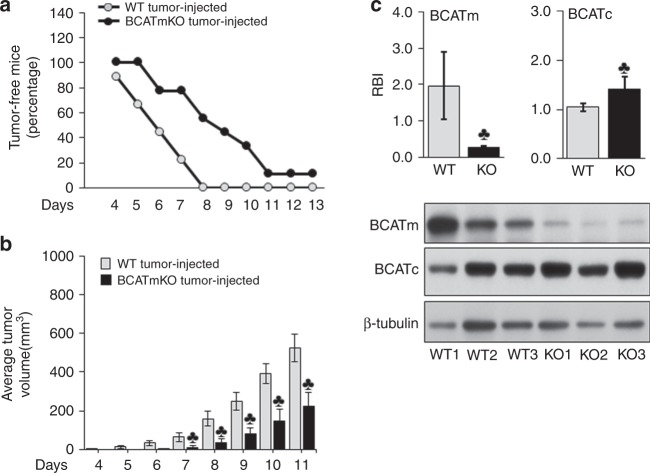
BCATmKO mice respond with delayed tumour growth and reduced tumour size to EL-4 lymphoma. WT and BCATmKO mice were s.c. injected with 100 µl PBS buffer with or without 5 × 10^5^ EL-4 cells 3 days after being offered standard rodent chow. The appearance of tumours was monitored daily and as soon as tumours were visible, they were measured for up to 13 days. **a** Tumour free mice. **b** Average tumour volume. **c** Protein expression of BCATm and BCATc in tumour tissues from three representative WT (WT1-3) and BCATmKO (KO1-3) mice. The average tumour expression of BCATm and BCATc was quantified by Image J and shown as relative band intensity (RBI). In all graphs, data represent mean ± SEM, *n* = 9, females, age 12-15 weeks, ^♣^*P* ≤ 0.05 as compared to tumour-injected WT mice

To determine whether global deletion of BCATm impacts the growth of lymphoma in mice, we subcutaneously injected EL-4 cells in the upper back of BCATmKO and WT mice. As shown in Fig. 1a, BCATmKO mice remained tumour free for a longer period as compared to WT mice. By day 6, 55.6% of the WT mice developed tumours compared to only 22.3% of BCATmKO mice. By day 8, all WT mice were tumour bearing, while 55.5% of BCATmKO mice were still tumour free. Of these, 11% never developed tumours (day 13, Fig. 1a). Moreover, BCATmKO mice developed tumours of smaller size (more than 50% reduction as compared to WT tumours, Fig. 1b). These results suggested that global deletion of BCATm had a protective anti-tumour effect.

The EL-4 cells were not genetically modified to delete the gene encoding BCATm, therefore, these cells expressed normal amounts of BCATm (Supplementary Figure 3B). However, due to the heterogenous nature of the tumour tissue, where the host organism contributed to the formation of the growing tumour, there was a substantial reduction by 86.5% in the expression of BCATm in tumours isolated from BCATmKO mice (Fig. 1c). The expression of BCATc, was significantly higher by 35% in tumour tissues from BCATmKO mice compared to WT mice (Fig. 1c). The significantly lower expression of BCATm, but higher BCATc, was suggestive of a compensatory mechanism to sustain BCAA metabolism in tumour tissues of BCATmKO mice.

### A loss of BCATm leads to a build-up of BCAAs in plasma and tumours

**Fig. 2:**
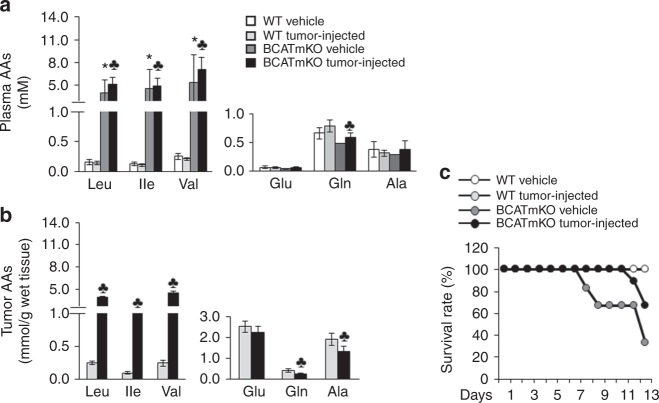
Tumour-bearing BCATmKO mice survive the toxic accumulation of BCAAs when fed standard rodent chow. WT and BCATmKO mice were challenged with EL-4 cells as described in Fig. 1. Amino acid (AA) concentrations of BCAAs (Leu, Ile, Val), glutamate (Glu), glutamine (Gln), and alanine (Ala) in plasma (**a**) and tumour tissues (**b**) were measured in the end of the tumour study (day 13). **c** Survival rates were measured every day until the end of the tumour study (day 13). Data represent mean ± SEM, *n* = 6-9, females, age 12-15 weeks, ^♣^*P* ≤ 0.05 as compared to tumour-injected WT mice; *P ≤ 0.05 as compared to vehicle WT mice

The standard rodent chow used during the tumour study, contained 1.8, 0.8, and 0.9 g/kg leucine, isoleucine, and valine, respectively (Supplementary Table 2). BCATmKO mice failed to metabolise the dietary BCAAs, resulting in a 35.8-, 44-, and 33.2-fold build-up of plasma leucine, isoleucine, and valine, respectively, compared to plasma BCAAs in WT mice (Fig. 2a). The development of tumours did not affect the plasma concentrations of BCAAs, as there were no significant differences between vehicle- and tumour-injected BCATmKO mice (Fig. 2a). In the tumour tissues of BCATmKO mice, the concentrations of leucine, isoleucine, and valine increased by a 15.6-, 23.2-, and 17.9-fold, respectively, compared to those in WT mice (Fig. 2b). Although the tumour BCAAs were not as high as the plasma BCAAs in BCATmKO mice, the elevated tumour expression of BCATc (Fig. 1c) was not sufficient to decrease tumour BCAAs in BCATmKO mice to those levels found in tumour-bearing WT mice. Since the BCAT enzymatic reaction produces glutamate, we looked at plasma and tumour concentrations of glutamate along with glutamine and alanine; the latter are synthesised from glutamate and pyruvate. Vehicle- and tumour-injected BCATmKO mice had lower, although not statistically different, concentrations of plasma glutamate compared to vehicle- and tumour-injected WT mice, respectively. Furthermore, plasma glutamine, but not alanine, concentrations were significantly lower in tumour-injected BCATmKO mice compared to tumour-injected WT mice (Fig. 2a). In tumour tissues of BCATmKO mice, the concentrations of glutamine and alanine were decreased by 37.5% and 30.4%, respectively, when compared to these amino acids in WT tumours; glutamate, however, did not show a statistically significant decrease (Fig. 2b). Other amino acids were also determined (Supplementary Tables 3 and 4) but were mostly unaffected. However, arginine and ornithine were lower in tumour tissues of BCATmKO mice (Supplementary Table 4). Because glutamate and glutamine are substrates for the synthesis of arginine and ornithine, the suppression of BCAA metabolism in BCATmKO mice may have had an impact on the synthesis of these amino acids. In conclusion, BCATmKO mice fed standard rodent chow experienced a dramatic build-up of BCAAs but decreased concentrations of glutamine, alanine, arginine, and ornithine in plasma and/or tumour tissues.

### Tumour formation has a protective effect on the survival of BCATmKO mice fed standard rodent chow

Consumption of standard rodent chow negatively impacted the survival of BCATmKO mice due to the inability to metabolise dietary BCAAs in the absence of BCATm (Fig. 2a).^25,35^ To explore whether tumour growth would affect the survival of BCATm mice fed standard rodent chow, the survival rates of vehicle- and tumour-injected BCATmKO mice and their WT counterparts were compared. Tumour-injected BCATmKO mice experienced a 67% survival rate in comparison to only a 33% survival rate of vehicle-injected BCATmKO mice (day 13, Fig. 2c). WT (vehicle- and tumour-injected) mice showed 100% survival rate, as expected. The beneficial effect of the growing tumour on the survival of BCATmKO mice was not due to lower food intake, as no differences in food intake or body weight were observed between tumour-injected and vehicle BCATmKO or WT mice (Supplementary Figure 1). Although the mechanism of this effect remains currently unknown, tumour formation alleviated the adverse effect of the standard rodent chow on the survival of BCATmKO mice.

### BCATmKO mice suffer from organ hypertrophy independent of tumour formation

**Fig. 3:**
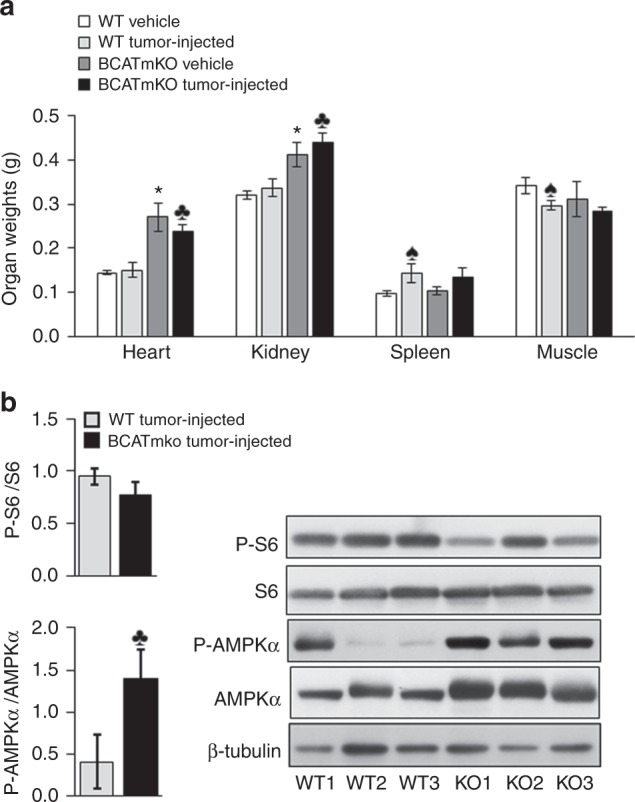
**a** Organ hypertrophy in BCATmKO mice. Heart, kidney, spleen, and muscle (gastric and soleus) weights are expressed in grams (g) and are adjusted for body weight (25 g). **b** Western blotting of S6 and AMPKα from tumour tissues. Representative protein images from three WT (WT1-3) and BCATmKO (KO1-3) mice are shown. Image J software was used to calculate the relative band ratio between the phosphorylated (P) and total forms of S6 and AMPKα. In all graphs, WT and BCATmKO mice were challenged with EL-4 cells as described in Fig. 1 and organs and tissues were harvested in the end of the tumour study (day 13). Data represent mean ± SEM, *n* = 6–9, females, age 12–15 weeks, ^♣^*P* ≤ 0.05 as compared to tumour-injected WT mice; *^♠^*P* ≤ 0.05 as compared to vehicle WT mice

Upon generation of the global BCATmKO mouse, it was discovered that male BCATmKO mice had an enlarged heart and kidneys, while other tissues were normal.^25^ The female BCATmKO mice used in this study also experienced hypertrophy of the heart and kidneys when fed standard rodent chow (Fig. 3a). Tumour development neither improved nor worsened organ hypertrophy, as no differences in organ weights were recorded between vehicle- and tumour-injected BCATmKO mice. Tumour formation had a slight impact on spleen and muscle as spleen enlargement, but muscle mass reduction, was identified in tumour-challenged mice as compared to vehicle-induced mice (Fig. 3a). However, no difference in spleen and muscle weights between tumour-injected WT and BCATmKO mice were observed, suggesting that the impact of tumour growth on these organs was independent from the global deletion of BCATm.

### AMPKα, but not mTORC1 signalling, is activated in tumour tissues of BCATmKO mice

Previous investigations revealed that disruption of BCATm resulted in increased energy expenditure and alterations in complex 1 of the mammalian target of rapamycin (mTORC1) and the AMP-regulated protein kinase (AMPK) in the heart, liver, and skeletal muscles.^25,35^ To test whether tumours isolated from BCATmKO mice fed standard rodent chow would show changes in mTORC1 signalling and AMPK, we measured the phosphorylation of S6, one of the downstream targets of mTORC1, as well as the total and phosphorylated forms of AMPKα in tumour tissues of BCATmKO and WT mice. The phosphorylation of S6 was slightly decreased in tumours of BCATmKO mice, but the difference was not significant (Fig. 3b). However, the phosphorylation of AMPKα was significantly increased by a 3.5-fold in BCATmKO tumours compared to WT tumours, which suggested higher activity of AMPKα and lower energy status of the BCATmKO tumours (Fig. 3b). Since leucine can be metabolised to acetyl-CoA and used for energy production, the substantial reduction of BCATm in BCATmKO tumours may have negatively impacted the energy status of the tumours, causing activation of AMPKα.

### Diet composition affects the ability of BCATmKO mice to fight tumour growth

**Fig. 4:**
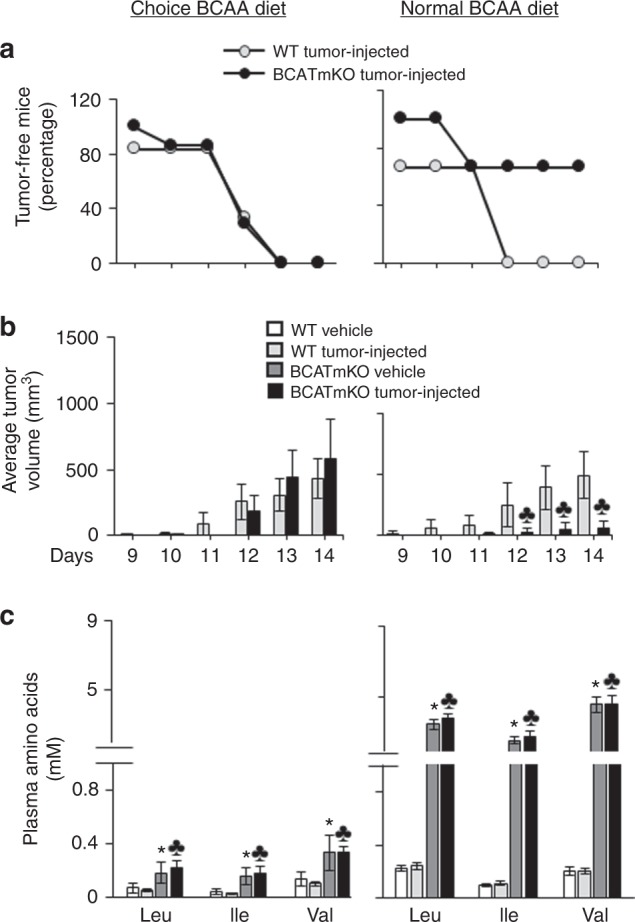
Diet composition impacts the ability of BCATmKO mice to fight tumour growth. Half of WT and BCATmKO mice remained on a choice BCAA diet, while the other half were offered a normal BCAA diet three days prior to s.c. injections with 2.5 × 10^5^ EL-4 cells. The appearance of tumours was monitored daily and as soon as tumours were visible (day 9), they were measured for up to 14 days. **a** Tumour-free mice. **b** Average tumour volumes. **c** Plasma concentrations of BCAAs (Leu, Ile, Val) as determined in the end of the tumour study (day 14). In all graphs, data represent mean ± SEM, *n* = 3–9, females, age 12–14 weeks, ^♣^*P* ≤ 0.05 as compared to tumour-injected WT mice; *P ≤ 0.05 as compared to vehicle WT mice

To survive, BCATmKO mice are maintained on a choice BCAA diet that prevents the accumulation of toxic amounts of BCAAs in BCATmKO mice.^25^ Consumption of standard rodent chow, however, led to toxic build-up of BCAAs and reduced mouse survival (refer to Fig. 2). This prompted us to explore whether offering a choice BCAA diet would prevent the negative impact of toxic accumulation of BCAAs and retain the ability of BCATmKO to fight tumour growth. We separated WT and BCATmKO mice into two groups, one on a choice BCAA diet (mix of normal and low BCAA diets) and the other on a normal BCAA diet only, followed by challenging the mice with EL-4 cells. WT mice (vehicle- and tumour-injected) preferably consumed a normal BCAA diet, while BCATmKO mice (vehicle- and tumour-injected) preferably consumed a low BCAA diet (Supplementary Figure 2A) as shown previously.^25^ However, there was no difference in total food intake or body weight between WT and BCATmKO mice on a choice BCAA diet (Supplementary Figure 2B, C). In contrast, BCATmKO mice consumed less food most days when offered a normal BCAA diet only and showed a slight reduction in body weight (Supplementary Figure 2B, C). Regarding tumour development, BCATmKO animals fed a choice BCAA diet lost their ability to remain tumour free and to fight tumour growth, as no difference in tumour development or tumour volume was observed between WT and BCATmKO mice (Fig. 4a, b). Additionally, no difference in plasma glutamate, glutamine, and alanine were observed (data not shown). In contrast, offering a normal BCAA diet contributed to the anti-tumour abilities of BCATmKO mice, where 66.6% of these mice remained tumour free (Fig. 4a). BCATmKO mice that developed tumours experienced more than 80% reduction in tumour volume in comparison to tumours developed by WT mice (Fig. 4b). Although BCATmKO mice fed a normal BCAA diet had similar changes in the expression of tumour BCATm and BCATc as BCATmKO mice on a choice BCAA diet (Supplementary Figure 2D), the diet composition determined the response of these animals to the growing lymphomas. The choice BCAA diet led to only a 2-fold increase in plasma BCAAs, which was not high enough to have protective effect on tumour growth in BCATmKO mice (Fig. 4c). In contrast, the average concentrations of BCAAs in BCATmKO mice fed a normal BCAA diet were increased by a 19-fold when compared to WT mice on the same diet (Fig. 4c). Thus, the dramatic build-up of BCAAs correlated with delayed tumour growth in BCATmKO mice.

### High leucine concentrations are not beneficial for the growth or the glycolytic metabolism of EL-4 cells

**Fig. 5:**
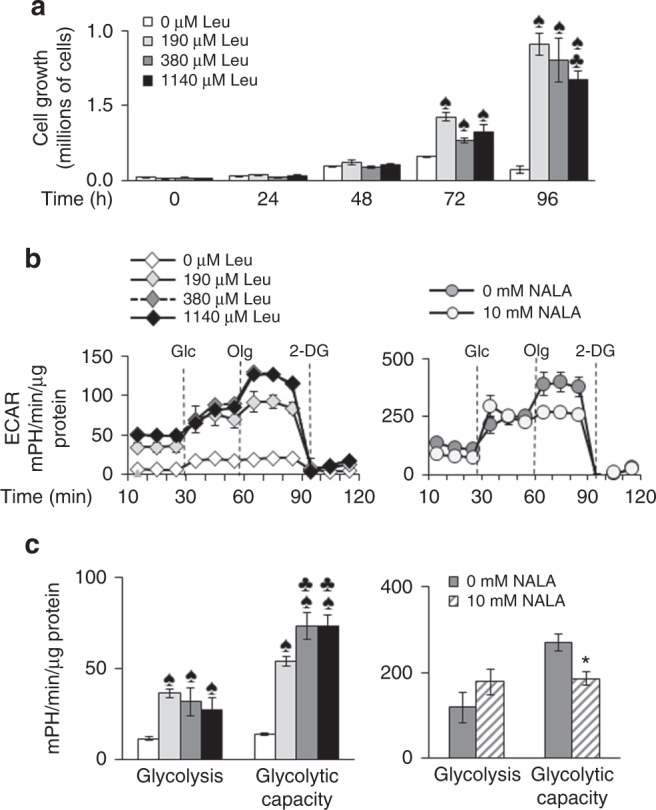
Impact of leucine on the growth and glycolytic metabolism of EL-4 cells. EL-4 cells were treated with increasing concentrations of leucine (0, 190, 380, and 1140 µM Leu) for 0–96 h or with 0 and 10 mM NALA for 24 h (see “Materials and methods” for details). **a** Cell growth as determined by the trypan blue exclusion method. **b** Glycolytic flux measured by the XF24 analyser. The extracellular acidification rate (ECAR) was measured in glucose-free medium, followed by addition of glucose (Glc). Next, oligomycin (Olg) was added to inhibit mitochondrial ATP synthesis, and 2-deoxy-d-glucose (2-DG) to inhibit glycolysis. **c** Glycolysis was calculated by subtracting the non-glycolytic ECAR from the glucose-induced ECAR, while glycolytic capacity was calculated by subtracting non-glycolytic ECAR from oligomycin-induced ECAR. Data represent mean ± SEM, *n* = 3 independent experiments. ^♠^*P* ≤ 0.05 as compared to 0 µM Leu. ^♣^*P* < 0.05 as compared to 190 µM Leu. *P < 0.05 as compared to 0 mM NALA

To understand why a build-up of BCAAs correlated with reduced tumour growth in BCATmKO mice, we studied the impact of one of the BCAAs (leucine) on the growth of EL-4 cells in vitro. As expected, leucine was necessary for growth of EL-4 cells, as adding 0 µM leucine to the growth medium gradually led to growth inhibition reaching 92% by 96 h (Fig. 5a). However, increasing leucine concentrations to 1140 µM led to a 25% reduction in EL-4 cell growth after 96 h. The most beneficial concentration of leucine was 190 µM, which was lower than the concentration used in the formulation of RPMI-1640 (380 µM leucine) (Fig. 5a). Thus, physiological (190 µM), but not high (1140 µM) concentrations of leucine promoted the growth of EL-4 cells.

Having in mind that amino acids are used as sources of carbon skeletons for glycolysis and other metabolic pathways, we next explored the effect of leucine on the metabolism of EL-4 cells.^36^ 0 µM of leucine, added to the growth medium caused a 67.6% reduction in the basal glycolysis compared to 190 µM leucine, the latter concentration led to the highest levels of basal glycolysis measured in EL-4 cells (Fig. 5b, c). Higher leucine concentrations failed to further increase the basal glycolytic rate but led to increased maximal glycolytic capacity (Fig. 5c). To confirm this observation, we used the leucine structural analog NALA to reduce leucine uptake. 10 mM NALA had an inhibitory effect on ECAR, following the addition of oligomycin, but not when glucose only was added. Thus, treatment with NALA did not decrease the basal glycolytic rate (observation seen before), but had an inhibitory effect on the maximal glycolytic capacity of EL-4 cells (Fig. 5b, c).^21^ Decreased glycolytic flux in EL-4 cells grown in medium supplemented with 0 µM leucine correlated with decreased activity of LDH-A and reduced concentrations of HEXII (Supplementary Figure 3A). However, high leucine concentrations (1140 µM) did not result in increased glycolytic activity, indicating that physiological, but not high, leucine concentrations were beneficial to glycolysis in EL-4 cells.

### Oxygen consumption and the energy status of EL-4 cells are dependent on the presence of leucine

**Fig. 6:**
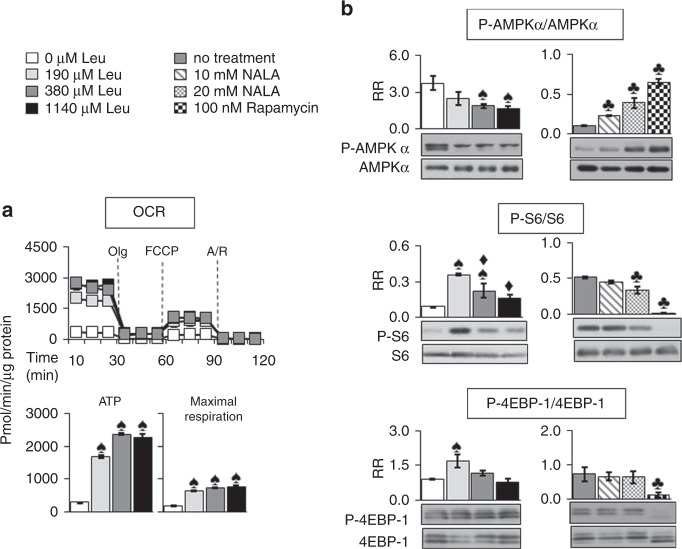
EL-4 cells treated with 0 µM leucine experience severe reduction in oxygen consumption, ATP production, and mTORC1 pathway. **a** Oxygen consumption rate (OCR) as measured by the XF24 analyser in EL-4 cells treated with increasing concentrations of leucine (0, 190, 380, and 1140 µM Leu) for 48 h. The basal respiration was measured first, followed by addition of oligomycin (Olg) to inhibit ATP synthesis, and addition of uncoupler (FCCP) of the oxidative phosphorylation. Lastly, antimycin and rotenone (A/R) were added to inhibit ETC and thus the mitochondrial respiration. The ATP production and maximal respiration were calculated as described.^34^ **b** Western Blotting of AMPKα, S6, and 4EBP-1 in EL-4 cells treated with leucine (0–1140 µM Leu) for 48 h, or NALA (0,10, 20 mM) and rapamycin (0, 100 nM) for 24 h. Image J software was used to calculate the relative ratio (RR) between the phosphorylated and total forms of AMPKα, S6, and 4EBP-1. In all graphs, data represent mean ± SEM, *n* = 3 independent experiments. ^♠^*P* ≤ 0.05 as compared to 0 µM Leu, ^♦^*P* ≤ 0.05 as compared to 190 µM Leu, ^♣^*P* < 0.05 as compared to no treatment

The energy-sensing metabolic regulator, AMPK, known to respond to falling ATP concentrations by reducing energy consuming pathways, was activated in tumours isolated from BCATmKO mice (refer to Fig. 3b).^37^ This observation prompted us to explore the oxidative metabolism of EL-4 cells, in which cells, the addition of 0 µM leucine to the growth medium severely reduced the oxygen consumption, the ATP production, and the maximal respiration of EL-4 cells compared to 190 µM or higher leucine concentrations (Fig. 6a). These results indicated that the energy status of EL-4 cells, supplemented with 0 µM leucine, was low. Notably, the energy sensor AMPKα was highly phosphorylated and thus activated by 0 µM leucine (a 2.2-fold increase as compared to 1140 µM leucine, Fig. 6b). Treatment of EL-4 cells with NALA also led to an increase in the phosphorylation of AMPKα (a 3.9-fold increase by 20 mM NALA, Fig. 6b), confirming that restriction of leucine availability negatively affected the energy status of EL-4 cells. Because EL-4 cells expressed normal amounts of BCATm, in contrast to tumours isolated from BCATmKO mice (refer to Fig. 1c and Supplementary Figure 3B), the EL-4 cells were capable of metabolising leucine. Therefore, providing these cells with higher leucine concentrations may have contributed to better energy status and reduced activity of AMPKα, respectively.

### mTORC1 signalling and apoptotic markers are affected by leucine availability in EL-4 cells

Leucine is one of the most potent activators of mTORC1 and we speculated that modulating the leucine concentrations in the growth medium of EL-4 cells would impact mTORC1 signalling.^26,38^ Addition of 0 µM leucine to the growth medium of EL-4 cells significantly reduced the phosphorylation of S6 and 4EBP-1 by 75% and 46%, respectively, when compared to EL-4 cells treated with 190 µM leucine (Fig. 6b). However, higher leucine concentrations (380 and 1140 µM) did not lead to higher S6 or 4EBP-1 phosphorylation when compared to 190 µM leucine, a finding that resembled the effect of leucine on the basal glycolytic rate (Fig. 5b, c). NALA (20 mM) significantly reduced the phosphorylation of S6 by 33%, which was not nearly as severe as the effect of rapamycin, a selective inhibitor of mTORC1 (Fig. 6b).^35^ The findings that high leucine concentrations were not beneficial for the growth, metabolism, and mTORC1 signalling of EL-4 cells suggested that the apoptotic program of these cells may have been affected. Indeed, a treatment with 1140 µM leucine decreased the concentrations of the anti-apoptotic marker Bcl-2 but increased the concentration of the pro-apoptotic marker BAX (Supplementary Figure 4A). Adding BAX-inhibiting peptide (BAX-V5) to EL-4 cells treated with 1140 µM leucine, reduced the protein expression of BAX by 40.2% as compared to cells grown in the presence of a negative control peptide (NCP) and 1140 µM leucine (Supplementary Figure 4B). Moreover, chromatin condensation was highest in EL-4 cells treated with 1140 µM leucine but was reduced by 42.4% when BAX-V5 was added (Supplementary Figure 5A). On the other hand, addition of etoposide, an activator of apoptosis, caused 84% or higher chromatin condensation in all EL-4 cells (Supplementary Figure 5A-C). These results suggested that high leucine concentrations did not prevent but enhanced EL-4 cell vulnerability to apoptosis, an effect that involved BAX- dependent mechanism.

## DISCUSSION

By taking advantage of the global knockout mouse model of BCATm, we now have evidence that BCATm is necessary for the progression of lymphoma, as global deletion of BCATm led to reduced lymphoma growth.^25^ While this was the first study to use an animal model of BCATm in cancer, other studies using siRNA to silence BCATm, or comparing the expression of BCATm in healthy and cancer tissues, also suggested that a loss of BCATm leads to suppression of tumour growth, while BCATm overexpression has the opposite effect.^23,24,39^ In this study, however, the diet composition significantly impacted the ability of BCATmKO mice to repress cancer. As previously reported, BCATmKO mice are maintained on a choice BCAA diet to escape the deleterious effects of toxic BCAA accumulation.^25^ Some of these effects are heart and kidney hypertrophies. Heart hypertrophy was previously linked with the well-established role of BCAAs, and particularly leucine, in the regulation of mTORC1 signalling.^23,24,39^ In fact, feeding BCATmKO mice with a diet supplemented with rapamycin prevented the heart enlargement of BCATmKO animals, suggesting that mTORC1 is the mediator of these effects.^35^

Consumption of a choice BCAA diet did not correlate with differences in tumour growth between WT and BCATmKO mice. In fact, BCATmKO mice could fight lymphoma only when fed diets that led to a dramatic build-up of BCAAs. Given that BCAAs are sources of metabolic fuel for the growth of proliferative cells, it was an unexpected discovery that the build-up of BCAAs in tumour tissues of BCATmKO mice would negatively impact tumour growth.^1,40^ Moreover, mTORC1 signalling, known to stimulate protein synthesis and other anabolic processes, was not upregulated by the highly elevated tumour BCAAs.^38^ Similarly, increased concentrations of BCAAs, but a lack of upregulation of mTORC1, were described in glioblastoma cells.^7^ In contrast, studies with breast cancer and CML blast crisis, showed that elevated concentrations of BCAAs correlated with upregulation of mTORC1 and increased tumour growth.^1^ Further studies with systemically administered BCAAs may be needed to fully address the functional connection between BCAAs and mTORC1 signalling in tumour growth.

In contrast to mTORC1 signalling, AMPK was activated in tumours of BCATmKO mice. Because AMPK is activated in response to low energy status, this finding suggested that tumour energy levels were negatively impacted by the severe reduction of BCATm expression.^41,42^ Blocked BCAA catabolism would decrease the production of energy intermediates, such as acetyl-CoA, which is a final product of leucine and isoleucine degradation.^43^ Maple Syrup Urine Disease (MSUD), an inherited disorder in the branched chain keto-acid dehydrogenase complex (BCKDC) that catalyses the second step in BCAA degradation, is also associated with a build-up of BCAAs.^44^ By using a mouse model of MSUD, Sonnet et al., demonstrated that the build-up of BCAAs is associated with an increase in muscle AMP.^45^ In response to increased AMP concentrations, AMPK is activated and directly phosphorylates a series of substrates (among them are Acetyl-CoA carboxylase and HMG-CoA reductase) while providing adaptive metabolic reprogramming through regulation of transcription factors, coactivators, and histone deacetylases.^37^ This ultimately causes upregulation of catabolism to generate energy in the form of ATP. Alternatively, AMPK switches off anabolic pathways to reduce energy consumption. One of the mechanisms by which AMPK regulates anabolic processes is suppression of mTORC1 pathway, which can occur by two different mechanisms: (1) activation of the tuberculosis sclerosis complex 2 (TS2), which in turn inhibits mTORC1 or by (2) direct phosphorylation and inhibition of mTORC1 at Thr2446.^41^ Because the role of AMPK is to inhibit pathways that lead to growth, AMPK can be regarded as a tumour suppressor. In the case of BCATmKO animals, it is possible to speculate that a blockage in tumour BCAA metabolism likely contributed to the activation of AMPK, which in turn prevented upregulation of mTORC1 and caused suppression of tumour growth. A recent study identified AMPK as an upstream regulator of BCATm.^15^ Activation of AMPK in PDAC cells, deficient of the mitochondrial malic enzyme 3 (ME3), suppressed BCATm gene expression. Conversely, pharmacological activation of AMPK by AICAR, or metformin, down-regulated the expression of BCATm.^15,45^ While the current study focuses on the global deletion of BCATm, it is intriguing to speculate that BCATm may be one of the downstream targets of AMPK.

Cancer cells maintain high glycolytic flux to support the increased biosynthetic demands upon oncogenic transformation.^46,47^ This is accompanied by increased activities of several glycolytic enzymes, including HEXII and LDH.^47,48^ One of the isoforms of LDH, LDH-A, is overexpressed in many cancers and is responsible for the large amounts of lactate secreted by cancer cells.^49^ Modulating the concentrations of leucine in the growth medium of EL-4 cells revealed that physiological concentrations of leucine were important for the maintenance of high glycolytic flux and up-regulation of HEXII and LDH-A. Because, the leucine transamination product α-ketoisocaproate can be converted into acetyl-CoA, which in turn is oxidised in the TCA cycle, the EL-4 cells might have become less dependent on glucose to generate acetyl-CoA thus diverting the glycolytic flux toward more lactate production.^1,22^ Supplying EL-4 cells with leucine concentrations, higher than physiological, led to higher glycolytic capacity and oxygen consumption; however high leucine concentrations failed to further stimulate lactate production (LDH activity was down-regulated) or cell growth suggesting that high leucine concentrations may exert a toxic effect on EL-4 cells.

Glutamate, the precursor of glutamine, is a side product of the BCAT enzyme reaction. Glutamine is an important source of nitrogen for amino acid and protein synthesis as well as de novo synthesis of nucleotides in cancer cells.^47,50^ The concentrations of glutamine, along with alanine and ornithine, were lower in tumours harvested from BCATmKO mice implying that the global deletion of BCATm had a negative impact on the non-essential amino acid synthesis in BCATmKO mice. Inhibition of amino group transfer from BCAAs to α-ketoglutarate and a blockage in nucleotide synthesis were reported in ME3-deleted PDAC cells in association with downregulation of BCATm expression.^15^ Similar findings were described in NSCLC, where extraction of nitrogen from BCAAs was important for de novo amino acid and nucleotide biosynthesis. The NSCLC tumours displayed increased expression of BCATm.^23^ These studies strongly suggest that BCAAs are important nitrogen sources for amino acid and nucleotide biosynthesis in cancer cells but cannot fully explain the correlation between the build-up of BCAAs and the reduction of tumour growth in BCATmKO mice, or the toxic growth effect of high leucine concentrations observed in EL-4 cells. A possible clue comes from the induction of apoptosis mediated by the pro-apoptotic marker BAX in the presence of high leucine concentrations. Supplying EL-4 cells with high leucine concentrations increased the expression of BAX and led to more chromatin condensation, this effect was reversed when BAX-V5 inhibitor was added. Our results were consistent with those reported by others where supplementation with BCAAs increased the levels of BAX in mouse livers and was proposed to play a role in the chemoprevention of liver tumourigenesis.^51^ Likewise, induction of apoptosis via BAX, following acute administration of BCAAs in rat hippocampus and cerebral cortex, was associated with the neurological effects of BCAAs seen in MSUD.^52^

In summary, our investigation unveiled a complex interplay between BCAA metabolism, glycolysis, amino acid synthesis, apoptosis, and the energy state of lymphoma tumours, while emphasising the important role of BCATm in regulating tumour BCAA concentrations. Furthermore, we showed that a build-up of BCAAs correlated with reduced lymphoma growth in a murine model. However, this study focused on analysing lymphoma growth on a whole organ level without taking into account the contributions of different cellular compartments within the tumour tissues. Because the tumour tissues are highly heterogenous, future directions aiming at deciphering the role of BCATm and BCAA metabolism in the tumour microenvironment will be explored. This will include investigations on how BCAA metabolism impacts the interactions between host (immune) and lymphoma cells and will ultimately benefit the development of new immunotherapeutic strategies to target lymphomas.

## Electronic supplementary material

Supplementary Information

Supplementary Figure 1

Supplementary Figure 2

Supplementary Figure 3

Supplementary Figure 4

Supplementary Table 1

Supplementary Table 2

Supplementary Table 3

Supplementary Table 4

Supplementary Figure 5
