# Supplementary material for: Mice deficient in the mitochondrial branched-chain aminotransferase (BCATm) respond with delayed tumour growth to a challenge with EL-4 lymphoma
Source: Br J Cancer. 2018 Oct 15;119(8):1009–17. doi: 10.1038/s41416-018-0283-7 (PMC6203766; doi:10.1038/s41416-018-0283-7)
Supplement: Supplementary file 1 — Supplementary Information [file 41416_2018_283_MOESM1_ESM.docx]

**Supplementary Table 1.** Composition of choice BCAA diet.

**Supplementary Table 2.** Standard rodent chow.

**Supplementary Table 3.** Plasma amino acid concentrations (µM).

**Supplementary Table 4.** Tumor amino acid concentrations (µmol/g wet tissue).

**Supplementary Figure 1. Food intake and body weights of BCATmKO mice on standard rodent chow.** WT and BCATmKO mice were challenged with EL-4 cells as described in Figure 1, average food intake (**A**), and body weight (**B**) were measured every other day until the end of tumor study (day 13). Data represent mean ± SEM, n=6-9, females, age 12-15 weeks.

**Supplementary Figure 2. Food intake, body weight, and tumor BCAT protein expression in mice fed choice and normal BCAA diets.** WT and BCATmKO mice were challenged with EL-4 cells as described in Figure 4. (**A**) Voluntary consumption of normal or low BCAA diets by mice offered a choice BCAA diet. The graph is representative of one day food intake (day 1). Similar consumption pattern was recorded for the remaining days of the tumor study. (**B**) Averaged food intake. For mice offered a choice BCAA diet, data represent combined consumption of normal BCAA and low BCAA diets. (**C**) Average body weight. Both food intake and body weight were measured every other day until day 13 of the tumor study. (**D**) Western blotting of BCATm and BCATc from tumor tissues. Representative protein images from three WT (WT1-3) and up to three BCATmKO (KO1-3) mice on the two different diets are shown. Note that there was only one tumor from BCATmKO mice fed a normal BCAA diet. The rest of the mice did not develop tumors. Image J software was used to calculate the relative band intensity of BCATm and BCATc after normalizing to β-tubulin. In all graphs, data represent mean ± SEM, n=5-9, females, age 12-14 weeks, ^♣^P≤ 0.05 as compared to tumor-injected WT mice; ^♠^P≤ 0.05 as compared to vehicle WT mice.

**Supplementary Figure 3. Leucine impacts the expression of metabolic proteins in EL-4 cells**. EL-4 cells were treated with increasing concentrations of leucine (0, 190, 380, and 1140 µM Leu) for 48 h, or NALA (0,10,20 mM), or rapamycin (0, 100 nM) for 24 h (see “Materials and Methods” for details) and the expression of glycolytic enzymes LDH-A and HEXII (**A**) and BCATm and BCATc (**B**) were determined by Western Blotting. Image J software was used to calculate the relative ratio (RR) between the phosphorylated and total forms of LDH-A or the relative band intensity (RBI) of BCATm and BCATc after normalizing to β-tubulin. Data represent mean ± SEM, n=3 independent experiments supported by representative protein images. ^♠^P≤ 0.05 as compared to 0 µM Leu, ^♦^P≤ 0.05 as compared to 190 µM Leu, ^♣^P<0.05 as compared to no treatment.

**Supplementary Figure 4. Leucine impacts the expression of apoptotic proteins in EL-4 cells**. The protein expression of Bcl-2 and BAX were determined with Western Blotting. Image J software was used to calculate the relative band intensity (RBI) of Bcl-2 and BAX after normalizing to β-tubulin. (**A**). Bcl-2 and BAX in EL-4 cells grown in medium supplemented with 0, 190, 380, or 1140 µM Leu, for 48 h, or NALA (0,10,20 mM), or rapamycin (0, 100 nM) for 24 h. (**B**). BAX in EL-4 cells grown with either 190 or 1140 µM Leu and simultaneously treated with 100 µM negative-control peptide (NCP), or 100 µM Bax-V5 for 48 h, or etoposide (30 µM) for 24 h. Data represent mean ± SEM, n=3 independent experiments supported by representative protein images. ^♠^P≤ 0.05 as compared to 0 µM Leu, ^♦^P≤ 0.05 as compared to NCP-1140 µM Leu, ^♣^P<0.05 as compared to no treatment. (see “Materials and Methods” for details).

**Supplementary Figure 5. Inhibition of BAX reduces apoptosis in EL-4 cells grown with high leucine concentrations.** Apoptosis was evaluated by measuring chromatin condensation in Hoechst-stained nuclei of EL-4 cells. (**A**). Average percentage of chromatin condensation in EL-4 cells supplemented with either 190 or 1140 µM Leu and simultaneously treated with negative-control peptide (NCP, 100 µM), or BAX-V5 (100 µM) for 48 h, or 30 µM etoposide for 24 h. Data represent mean ± SEM, n=3. ^♠, ♣^ P≤ 0.05 as compared to NCP-190 or NCP-1140 µM Leu, respectively, ^♦^P≤ 0.05 as compared to NCP-190 µM Leu. (**B-C**). Representative images (x10 magnification), taken under phase (Phase) and fluorescent (Hoechst) light of EL-4 cells supplemented with 190 µM (**B**) or 1140 µM Leu (**C**) and subjected to NCP, BAX-V5, or etoposide treatments (see “Materials and Methods” for details).
