# Supplementary figures and images for: Mice deficient in the mitochondrial branched-chain aminotransferase (BCATm) respond with delayed tumour growth to a challenge with EL-4 lymphoma
Source: Br J Cancer. 2018 Oct 15;119(8):1009–17. doi: 10.1038/s41416-018-0283-7 (PMC6203766; doi:10.1038/s41416-018-0283-7)

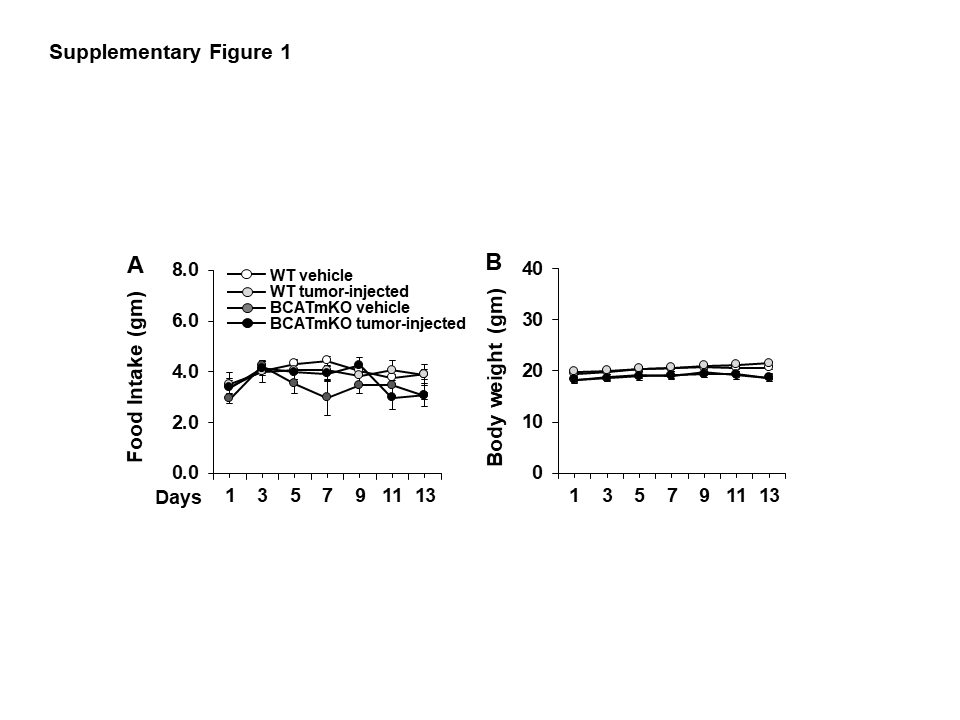

Supplement: Supplementary file 2 — Supplementary Figure 1 [file 41416_2018_283_MOESM2_ESM.tif]

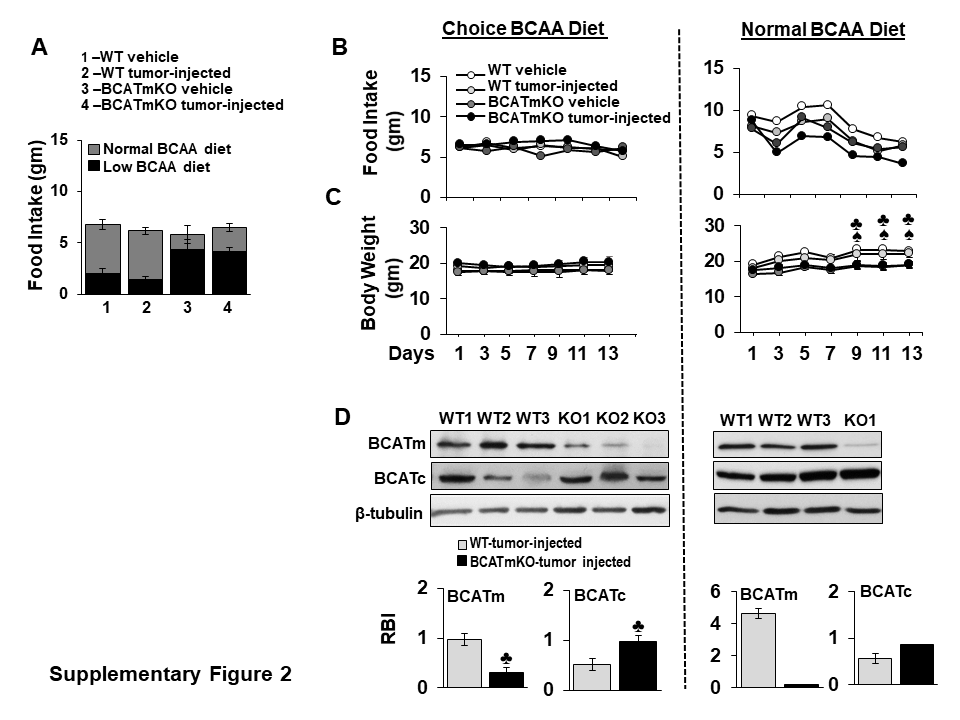

Supplement: Supplementary file 3 — Supplementary Figure 2 [file 41416_2018_283_MOESM3_ESM.tif]

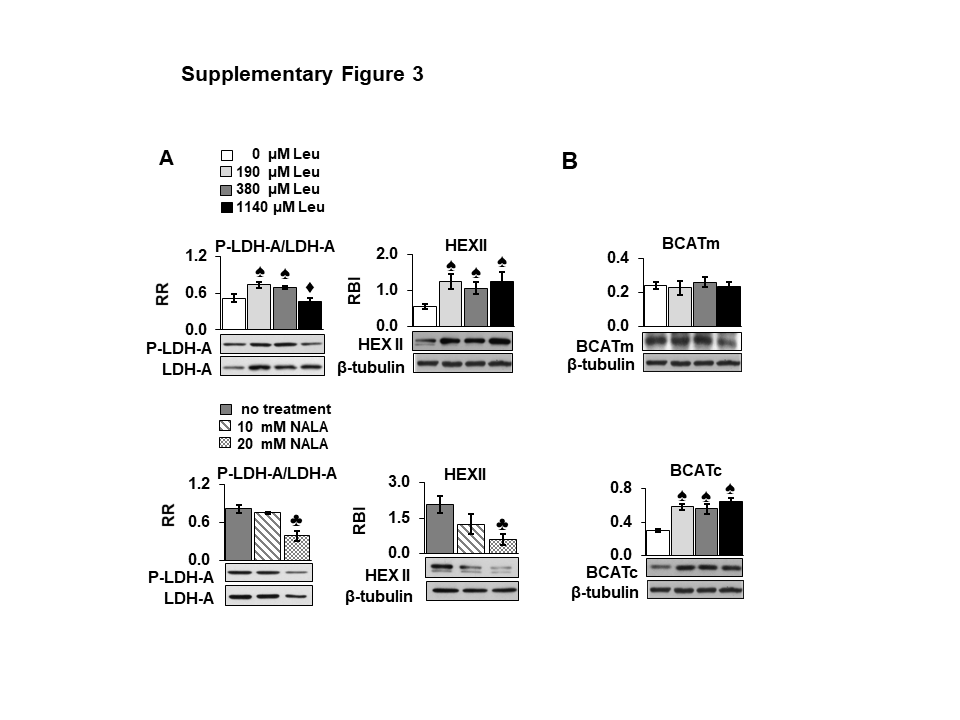

Supplement: Supplementary file 4 — Supplementary Figure 3 [file 41416_2018_283_MOESM4_ESM.tif]

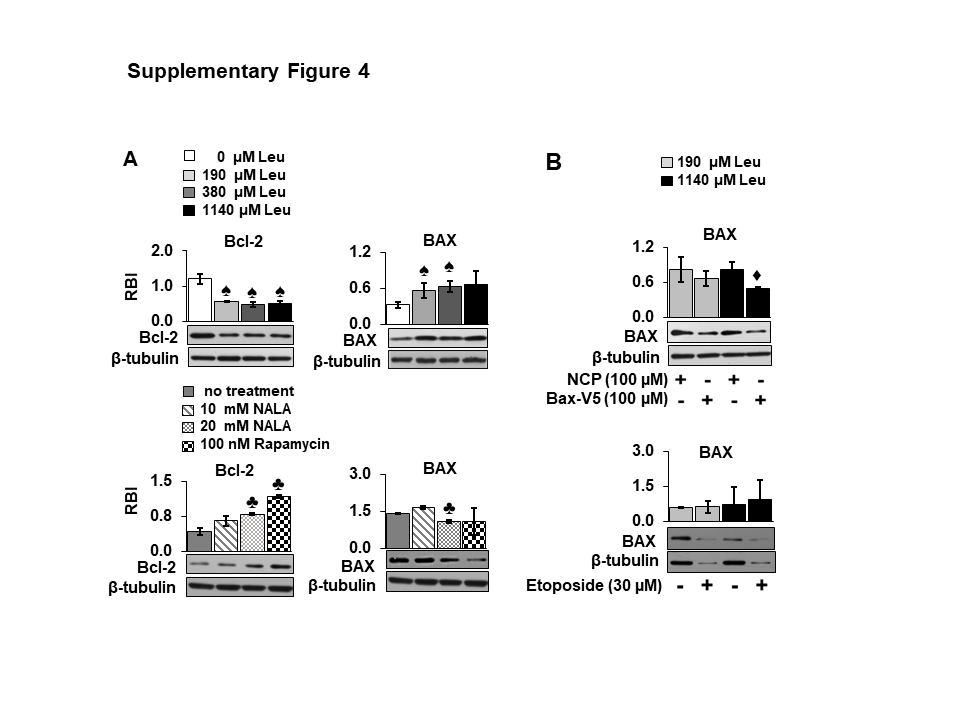

Supplement: Supplementary file 5 — Supplementary Figure 4 [file 41416_2018_283_MOESM5_ESM.tif]

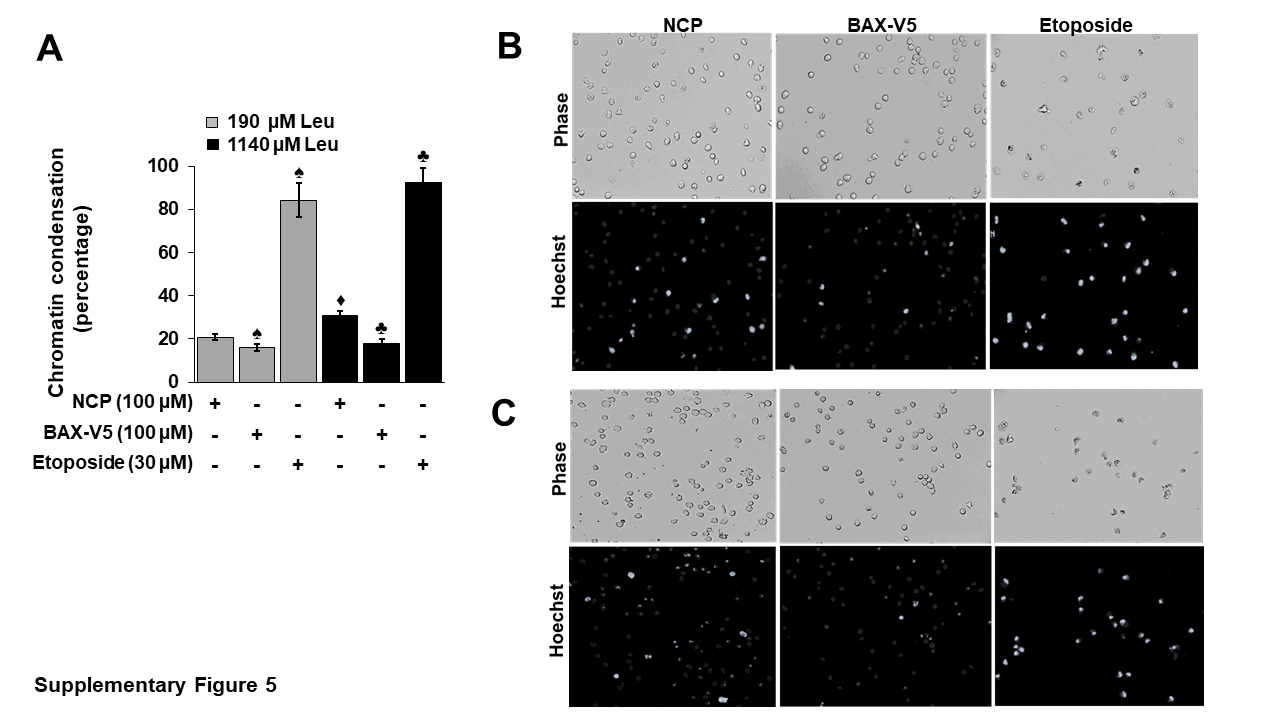

Supplement: Supplementary file 10 — Supplementary Figure 5 [file 41416_2018_283_MOESM10_ESM.tif]
