# Supplementary material for: Mice deficient in the mitochondrial branched-chain aminotransferase (BCATm) respond with delayed tumour growth to a challenge with EL-4 lymphoma
Source: Br J Cancer. 2018 Oct 15;119(8):1009–17. doi: 10.1038/s41416-018-0283-7 (PMC6203766; doi:10.1038/s41416-018-0283-7)
Supplement: Supplementary file 6 — Supplementary Table 1 [file 41416_2018_283_MOESM6_ESM.docx]

| **SUPPLEMENTARY TABLE 1. Composition of the choice BCAA diet^1^** | | | | |
| --- | --- | --- | --- | --- |
| **Diet** | **Normal BCAA diet** | | **Low BCAA diet** | |
|  | gm (%) | kcal (%) | gm (%) | kcal (%) |
| Protein | 16.0 | 16.6 | 13.3 | 14.1 |
| Carbohydrate | 69.3 | 71.7 | 69.6 | 73.8 |
| Fat | 5.1 | 11.8 | 5.1 | 12.1 |
| **Total** |  | **100.0** |  | **100.0** |
| **kcal/gm** | **3.87** |  | **3.77** |  |
|  |  |  |  |  |
| **Ingredients** | **gm** | **kcal** | **gm** | **kcal** |
| L-Arginine | 8.27 |  | 8.27 |  |
| L-Histidine-HCl-H_2_O | 6 |  | 6 |  |
| L-Isoleucine | 8 |  | 0.205 |  |
| L-Leucine | 12 |  | 0.278 |  |
| L-Valine | 8 |  | 0.205 |  |
| L-Lysine-HCl | 14 |  | 14 |  |
| DL-Methionine | 6 |  | 6 |  |
| L-Phenylalanine | 6 |  | 6 |  |
| L-Threonine | 8 |  | 8 |  |
| L-Tryptophan | 2 |  | 2 |  |
| L-Alanine | 10 |  | 10 |  |
| L-Asparagine-H_2_O | 10 |  | 10 |  |
| L-Aspartate |  |  |  |  |
| L-Cystine | 4 |  | 4 |  |
| L-Glutamic Acid | 10 |  | 10 |  |
| L-Glutamine | 10 |  | 10 |  |
| Glycine | 10 |  | 10 |  |
| L-Proline | 10 |  | 10 |  |
| L-Serine | 10 |  | 10 |  |
| L-Tyrosine | 10 |  | 10 |  |
| **Total L-Amino Acids** | **158.27** | **633.08** | **130.958** | **523.832** |
|  |  |  |  |  |
| Corn starch | 300 | 1200 | 300 | 1200 |
| Maltodextrin 10 | 125 | 500 | 125 | 500 |
| Sucrose | 250 | 1000 | 250 | 1000 |
| Cellulose | 50 | 0 | 50 | 0 |
| Soybean Oil | 50 | 450 | 50 | 450 |
| Mineral mix S 10001 | 35 | 0 | 35 | 0 |
| Sodium Bicarbonate | 7.5 | 0 | 7.5 | 0 |
| Vitamin Mix V 10001 | 10 | 40 | 10 | 40 |
| Choline Bitartrate | 2 | 0 | 2 | 0 |
| Diammonium citrate | 0 | 0 | 24 | 0 |
|  |  |  |  |  |
| FD&C Yellow Dye #5 | 0.05 | 0 | 0 | 0 |
| FD&C Red Dye #40 | 0 | 0 | 0.05 | 0 |
|  |  |  |  |  |
| **Total** | **987.8** | **3823.1** | **984.5** | **3713.8** |
|  |  |  |  |  |
| Na (gm) | 4.5 |  | 4.5 |  |
| Nitrogen (gm) | 22.1 |  | 22.1 |  |
| ^1^Choice BCAA diet constitutes of two diets: the normal and the low BCAA diets that were custom made by Research Diets Inc. | | | | |
