# Supplementary material for: Mice deficient in the mitochondrial branched-chain aminotransferase (BCATm) respond with delayed tumour growth to a challenge with EL-4 lymphoma
Source: Br J Cancer. 2018 Oct 15;119(8):1009–17. doi: 10.1038/s41416-018-0283-7 (PMC6203766; doi:10.1038/s41416-018-0283-7)
Supplement: Supplementary file 7 — Supplementary Table 2 [file 41416_2018_283_MOESM7_ESM.docx]

| **SUPPLEMENTARY TABLE 2. Standard rodent chow (Teklad Global 18% protein rodent Diet 2018)^1^** | | |
| --- | --- | --- |
| **Diet** | **Standard Rodent Chow** | |
| **Macronutrients** | **gm (%)** | **kcal (%)** |
| Crude Protein | 18.6 | 24 |
| Carbohydrate | 44.2 | 58 |
| Fat | 6.2 | 18 |
| Crude Fiber | 3.5 |  |
| Neutral detergent fiber | 14.7 |  |
| Ash | 5.3 |  |
|  |  |  |
| **Amino Acids** | **percent (%)** |  |
| L-Arginine | 1.0 |  |
| L-Histidine | 0.4 |  |
| L-Isoleucine | 0.8 |  |
| L-Leucine | 1.8 |  |
| L-Valine | 0.9 |  |
| L-Lysine | 0.9 |  |
| DL-Methionine | 0.4 |  |
| L-Phenylalanine | 1.0 |  |
| L-Threonine | 0.7 |  |
| L-Tryptophan | 0.2 |  |
| L-Alanine | 1.1 |  |
| L-Asparagine | 0 |  |
| L-Aspartate | 1.4 |  |
| L-Cystine | 0.3 |  |
| L-Glutamic Acid | 3.4 |  |
| L-Glutamine | 0 |  |
| Glycine | 0.8 |  |
| L-Proline | 1.6 |  |
| L-Serine | 1.1 |  |
| L-Tyrosine | 0.6 |  |
|  |  |  |
| **Minerals** | **mg/kg** |  |
| Zinc | 70 |  |
| Manganese | 100 |  |
| Copper | 15 |  |
| Iodine | 6 |  |
| Iron | 200 |  |
| Selenium |  |  |
|  | **percent (%)** |  |
| Calcium | 1.0 |  |
| Phosphorus | 0.7 |  |
| Non-Phytate Phosphorus | 0.4 |  |
| Sodium | 0.2 |  |
| Potassium | 0.6 |  |
| Chloride | 0.4 |  |
| Magnesium | 0.2 |  |
| ^1^Data was obtained from Harlan Laboratories website. | | |
