# Supplementary material for: Mice deficient in the mitochondrial branched-chain aminotransferase (BCATm) respond with delayed tumour growth to a challenge with EL-4 lymphoma
Source: Br J Cancer. 2018 Oct 15;119(8):1009–17. doi: 10.1038/s41416-018-0283-7 (PMC6203766; doi:10.1038/s41416-018-0283-7)
Supplement: Supplementary file 8 — Supplementary Table 3 [file 41416_2018_283_MOESM8_ESM.docx]

|  |
| --- |

| **SUPPLEMENTARY TABLE 3. Plasma amino acid concentrations (µM)** | | | | |
| --- | --- | --- | --- | --- |
| **WT** **BCATmKO**  **vehicle**  **vehicle** | | | **WT tumor- injected** | **BCATmKO tumor-**  **injected** |
| **Arginine** | nd | nd | nd | nd |
| **Histidine** | nd | nd | nd | nd |
| **Lysine** | 160 ± 30 | 149 ± 48 | 165 ± 26 | 254 ± 72 |
| **Methionine** | 47 ± 12 | 42 ± 17 | 37 ± 6 | 46 ± 10 |
| **Phenylalanine** | nd | nd | nd | nd |
| **Threonine** | 229 ± 75 | 197±129 | 170 ± 40 | 262 ± 68 |
| **Tryptophan** | 61 ± 10 | 73 ± 28 | 57 ± 9 | 58 ± 11 |
|  |  |  |  |  |
| **Asparagine** | 82 ± 29 | 84 ± 67 | 86 ± 15 | 153 ± 54 |
| **Aspartate** | 44 ± 12 | 42 ± 60 | 50 ± 7 | 50 ± 10 |
| **Cysteine** | nd | nd | nd | nd |
| **Glycine** | 195 ± 34 | 170 ± 00 | 168 ± 25 | 195 ± 43 |
| **Proline** | nd | nd | nd | nd |
| **Serine** | 70 ± 27 | 107 ± 00 | 72 ± 10 | 85 ± 23 |
| **Tyrosine** | 44 ± 26 | 54 ± 35 | 42 ± 15 | 44 ± 18 |
| **Ornithine** | 260 ± 30 | 312 ± 36 | 392 ± 116 | 422 ± 67 |
| **Taurine** | nd | nd | nd | nd |
| **Citrulline** | 96 ± 25 | 78 ± 37 | 74 ± 13 | 170 ± 28* |
| Plasma amino acid concentrations were determined in blood samples collected at the end of the tumor study (day 13) from WT and BCATmKO mice, that were either vehicle- or tumor-injected as described in Figure 1. All animals were fasted for 12 h prior to drawing blood for analysis (see Methods). Data represents mean ± SEM, n=6-9 female mice. **P*≤0.05 as compared to WT tumor-injected mice. | | | | |
