# Supplementary material for: Mice deficient in the mitochondrial branched-chain aminotransferase (BCATm) respond with delayed tumour growth to a challenge with EL-4 lymphoma
Source: Br J Cancer. 2018 Oct 15;119(8):1009–17. doi: 10.1038/s41416-018-0283-7 (PMC6203766; doi:10.1038/s41416-018-0283-7)
Supplement: Supplementary file 9 — Supplementary Table 4 [file 41416_2018_283_MOESM9_ESM.docx]

| **SUPPLEMENTARY TABLE 4. Tumor amino acid concentrations (µmol/g wet tissue)** | | |
| --- | --- | --- |
| **WT BCATmKO**  **tumor-injected tumor-injected** | | |
| **Arginine** | 32 ± 3 | 21 ± 1* |
| **Histidine** | nd | nd |
| **Lysine** | 666 ± 95 | 419 ± 144 |
| **Methionine** | 75 ± 7 | 173 ± 61* |
| **Phenylalanine** | 114 ± 19 | 163 ± 52 |
| **Threonine** | nd | nd |
| **Tryptophan** | 33 ± 5 | 35 ± 9 |
|  |  |  |
| **Asparagine** | 244 ± 41 | 253 ± 57 |
| **Aspartate** | 296 ± 76 | 297 ± 56 |
| **Cysteine** | nd | nd |
| **Glycine** | 2461± 234 | 2222 ± 584 |
| **Proline** | nd | nd |
| **Serine** | 445 ± 48 | 435 ± 69 |
| **Tyrosine** | 148 ± 24 | 170 ± 55 |
| **Ornithine** | 127 ± 23 | 109 ± 36* |
| **Taurine** | 6035 ± 652 | 3988 ± 784 |
| **Citrulline** | 76 ± 44 | 314 ± 65* |
| Tumor tissues from WT and BCATmKO mice, fed standard rodent chow, were collected in the end of the tumor study and used in an HPLC assay (see Materials and Methods). Data represents mean ± SEM, n=6 female mice, *P≤0.05 as compared to tumor-injected WT mice. | | |
